# Cardiovascular Health and Financial Hardship: Protocol for a Qualitative Citizen Science Study

**DOI:** 10.2196/89101

**Published:** 2026-03-13

**Authors:** Dagmar Niewold, Evy C E van Gestel, Latifa Abidi, Klaske Tiemstra, Myrte Thoolen, Gera E Nagelhout

**Affiliations:** 1 Independent scholar, Amersfoort, Utrecht, The Netherlands; 2 Centre of Expertise Perspective in Health, Avans University of Applied Sciences, Lovensdijkstraat 63, Breda, North Brabant, 4818 AJ, The Netherlands, 31 885256414; 3 Department of Health Promotion (CAPHRI), Maastricht University, Maastricht, Limburg, The Netherlands

**Keywords:** cardiovascular disease, citizen science, socioeconomic status, qualitative research, women’s health

## Abstract

**Background:**

Cardiovascular disease (CVD) is the leading cause of death worldwide. Individuals with lower income or experiencing financial hardship face a significantly higher risk of developing CVD. However, there is a lack of in-depth insight into their experiences with CVD, and specific attention to women is essential.

**Objective:**

The In a Heartbeat study aims to understand the relationship between CVD and financial hardship and enable earlier recognition and prevention of CVD among both women and men. In this study protocol, we describe our citizen science study, in which we unravel the mechanisms and contexts through which financial problems lead to the development and late recognition of CVD.

**Methods:**

The main data for this study are collected by citizen scientists through qualitative lifeline interviews. All citizen scientists have experience with financial hardship, and some also have experience with CVD. We hold weekly project team meetings with citizen scientists and professional scientists in which we use methods such as photo elicitation, critical-creative hermeneutic analysis, design thinking, a dynamic learning agenda, and regular individual and group evaluations of the citizen science process.

**Results:**

The study was funded in October 2024 and started in January 2025. Data collection started in November 2025 and is expected to end halfway through 2026. Four qualitative lifeline interviews had been conducted as of December 6, 2025. Data analyses are planned for 2026. Manuscripts reporting findings on the central research question and the process evaluations will be submitted for publication in 2027.

**Conclusions:**

Toward the end of the study in 2027, we will develop and disseminate concrete recommendations for various stakeholders to prevent CVD and recognize CVD earlier among people with financial hardship. In all our analyses and recommendations, we will consider sex and gender differences. Our study could contribute to a reduction in health inequalities.

## Introduction

Cardiovascular disease (CVD) is the leading cause of death worldwide. In 2019, an estimated 17.9 million people died from CVD, accounting for 32% of all global deaths. Of these, 85% were due to myocardial infarction and stroke [1]. In addition, looking at global causes of death from noncommunicable diseases among individuals younger than 70 years, 38% were attributed to CVD in 2019 [2]. Individuals with a lower socioeconomic position (SEP) face a significantly higher risk of developing CVD than those with a higher SEP worldwide [3,4]. This pattern is consistent across world regions, with approximately 80% of CVD deaths occurring in low- and middle-income countries [1]. Additionally, studies in both Australia and the Netherlands show that people in areas or groups with the lowest SEP have myocardial infarction and stroke rates 1.5 to nearly 2 times higher than those in areas or groups with the highest SEP [5-10]. Looking at socioeconomic differences in CVD death rates, the number of deaths attributed to CVD is higher for households with lower financial prosperity [11].

Several mechanisms explain the link between a lower SEP, lower income, or financial hardship and increased CVD risk. First, chronic psychosocial stress due to financial hardship represents an important mechanism leading to increased risk of CVD [12,13]. Financial insecurity, debt, unemployment, and social isolation cause chronic stress, which elevates stress hormones such as cortisol, triggers inflammatory responses, and results in vascular damage [14,15]. This chronic stress activates both the immune system and the hypothalamic-pituitary-adrenal axis, leading to chronic inflammation, hypertension, insulin resistance, abdominal fat accumulation, and other metabolic dysfunctions—all of which are known contributors to CVD development and progression [13,15,16].

Second, behavioral factors can play a role. Financial scarcity affects decision-making capacity by reducing cognitive bandwidth, forcing individuals to prioritize immediate needs while limiting resources for health-promoting behaviors. This scarcity mindset creates trade-offs between essential expenses and health care, contributing to treatment nonadherence and delayed care seeking [17]. Financial hardship restricts healthy choices and increases the likelihood of unhealthy behaviors such as tobacco use, poor diet, physical inactivity, and medication nonadherence, often used as coping mechanisms [8].

Third, limited access to care is a possible mediator between low income or financial hardship and CVD risk. Financial barriers hinder access to preventive care, necessary medications, treatments, and rehabilitation after cardiovascular events [8,18]. Finally, environmental factors can play a role. People with low incomes are more likely to live in environments with more stressors, such as food deserts, social isolation, and lack of safe spaces for physical activity, perpetuating unhealthy behaviors [10]. All the aforementioned factors can interact and reinforce each other, collectively increasing the risk and severity of CVD [8,18,19].

Although statistical links between SEP and CVD are well described and some of the mechanisms are known, there is a lack of in-depth understanding of the lived experiences and stories of people with CVD who have difficulty making ends meet. This is supported by a review by Powell-Wiley et al [20], which recommended future research to “incorporate qualitative approaches to better understand how individual lived experiences of marginalization affect cardiovascular health outcomes.” Specifically, little is known about how these individuals perceive and manage their health daily or how various contextual factors and mechanisms interact. Research on CVD recognition among women with financial difficulties is also scarce. Women have a poorer prognosis after cardiovascular events and experience higher postevent mortality rates. Additionally, women more frequently present with atypical symptoms (eg, extreme fatigue, shortness of breath, or dizziness) that are recognized later, contributing to delayed diagnosis and treatment [21,22]. These symptoms can resemble emotional and mental stress caused by financial hardship. Stress, a risk factor for CVD, can hinder symptom recognition, making women in this group particularly vulnerable to late diagnosis [8,23]. Finally, factors such as gender discrimination, a greater socioeconomic burden, and constraints on physical mobility among women may reduce their access to optimal cardiovascular care [22].

In this study, we focus on an underresearched group of people in a vulnerable position with an increased risk of CVD: people who have difficulty making ends meet. Our study is developed and conducted in cocreation with citizen scientists who have personal experience with financial hardship and/or CVD. Their experiential knowledge enriches all phases of the research, ensuring better alignment with the lived reality and needs of this group [24]. This participatory approach aligns with established frameworks for community health promotion that emphasize the importance of involving people with lived experience as equal partners in research [25]. This cocreation process increases the relevance of the research questions and the practical applicability of the results.

This study is conducted in the Netherlands. In 2024, almost half of Dutch households were classified as financially unhealthy or vulnerable, which is reflected in a lower healthy life expectancy for this group [26]. In the Netherlands, 23% of deaths in 2022 were attributed to CVD [27]. The aim of the In a Heartbeat study is to recognize CVD earlier and reduce CVD among people who have difficulty making ends meet. Our central research question is, How and in what groups does CVD develop among people who have difficulty making ends meet? Our research subquestions are (1) How can CVD be recognized early in this group, and what are the differences between women and men? (2) What are the best approaches to prevent CVD in this group, and do these differ for women and men? Answering these questions will result in developing concrete recommendations for various stakeholders, namely, people who have difficulty making ends meet themselves, health care and social professionals, health promoters, and policymakers. For all recommendations, we consider sex and gender differences. Consequently, these recommendations could contribute to a reduction in health inequalities.

## Methods

### Citizen Science

The study project team consists of 9 citizen scientists and 5 professional scientists working together as equals from the start. Eight citizen scientists are involved in an existing citizen advisory group, and 1 (the first author of this paper) is a certified expert by experience in poverty and health who also serves as co–project leader. All 9 citizen scientists have or have had experience with financial hardship, and some also have experience with CVD. The project team is supported by a supervisory committee with expertise in cardiology, sex and gender differences, citizen science, and socioeconomic health disparities. All citizen scientists are involved in every research phase as the entire process is carried out in cocreation. The collaboration is based on equality, open communication, and flexibility. This collaborative model follows principles of participatory systems approaches that have proven effective in community health promotion with people in a vulnerable position, emphasizing flexibility and shared decision-making [25]. We also follow the 10 principles of citizen science from the European Citizen Science Association [28]. Every project team member is paid for the time that they contribute to the project through an employment contract, a self-employed arrangement, or a volunteer allowance. The study duration is 2.5 years, and the study started in January 2025.

### Research Design

The research focuses on unraveling the mechanisms and contexts through which financial problems lead to the development of CVD, with special attention to sex and gender, health behaviors, and mental health. The main data for this study are collected through qualitative lifeline interviews. Additionally, we have weekly project team meetings in which methods such as photo elicitation, critical-creative hermeneutic analysis, design thinking, a dynamic learning agenda, and regular evaluations are used.

### Qualitative Interviews

We aim to conduct 40 to 60 interviews with adults (≥18 years) who have difficulty making ends meet (self-report) and have CVD (diagnosis). At recruitment, we ask potential participants whether they have difficulty making ends meet (“moeite met rondkomen” in Dutch) and whether they have a diagnosis of CVD (including high blood pressure or high cholesterol). Within the study population, the aim is to have an equal number of women and men and diversity in age, marital status, educational level, and cultural background. Interviewees are recruited through networks of citizen scientists and professional scientists, through relevant organizations, with the help of media attention for our study, and also through snowball sampling.

Interviews are conducted in duos consisting of a professional scientist and a citizen scientist or 2 citizen scientists. During the preparation phase of the study, citizen scientists receive interview training until they are confident enough to perform the interviews. This training includes general conversation techniques and concludes with practice interviews with mock interviewees.

Interviews take place at a location of choice by the interviewee, which can be at their home or, for example, at a community center or the university. Interviewees complete a short questionnaire including demographic characteristics (gender, age, educational level, household composition, and employment situation), their health situation (blood pressure, presence of diabetes, height, weight, and loneliness), lifestyle behaviors (tobacco use, alcohol use, and sedentary behavior), and financial situation (using the Psychological Inventory of Financial Scarcity [29]). During the interview, a timeline of life events related to CVD and financial challenges is created by one of the interviewers based on the input of the interviewee. On this “lifeline,” interviewees describe both their difficulty making ends meet and their CVD severity on a scale from 0 to 10, spanning from birth to the present. This timeline approach was chosen to specifically examine the relationship and trajectories between financial difficulties and CVD over time. The timeline allows for a retrospective lens to elicit autobiographical data covering personal disease trajectories [30]. The topic list includes questions about the financial situation of the interviewees, their experiences with CVD, health behaviors, sex- and gender-specific risks, mental health, living environment, and their interactions. The interviews are estimated to take 1 to 1.5 hours to complete. After the interview, emotional support for interviewers and interviewees is available when needed. This is provided by the expert by experience in the team and, if necessary, a psychologist.

### Photo Elicitation

Photo elicitation is a technique in which photographs are incorporated into research interviews [31]. Including photographs during interviews can lead to deeper reflections about participants’ experiences than what would be achieved through verbal questions [32]. It also helps build trust and improve communication during interviews, especially when experiences are emotionally charged [33]. The process to perform photo elicitation varies between studies as it is possible for interviewees as well as researchers to generate the photographs used [34].

Within this project, photo elicitation is used as both an interview and analysis technique. For the first 20 interviews, citizen scientists conducting the interviews take one or more photographs after each interview that capture their interpretation of the experiences of the interviewees. These photographs are discussed within the project team and used in data analysis. After the first 20 interviews, we decide whether it is feasible to add photo elicitation to the subsequent interviews, in which the photographs of the citizen scientists can serve as prompts for discussion with interviewees. This decision depends on how the previous interviews went, the length of these interviews, the quality of data collection thus far, and whether citizen scientists feel comfortable using photographs during interviews.

The goal of this approach is to enrich the data collection and analyses by using photographs. From a practical standpoint, on the one hand, our process minimizes the burden on interviewees (because they do not have to take the photos) while still incorporating photos that seek to capture the essence of the conducted interviews and, on the other hand, ensures that the photographs can be validated by subsequent participants.

### Data and Analysis

Qualitative interviews are transcribed and rewritten into anonymized readable stories, and the lifelines are digitized and anonymized. The stories and lifelines are checked by the interviewees before they are used in the analysis. The analysis is performed by the entire research team and follows the critical-creative hermeneutic framework by Cardiff and van Lieshout [35]. After the first interviews are performed, everyone reads or listens to the first anonymized stories and examines the lifelines and photographs. During creative analysis sessions, citizen scientists and professional scientists each make a creative display of the most important elements that they see in the stories, lifelines, and photos. Subsequently, they show their creative displays to the team and search for recurring elements in them, which they code. Phases of analysis are followed by, and inform, subsequent phases of interviewing in an iterative process.

After the creative analysis sessions, the original transcripts are used for thematic coding [36] based on the codes made during the joint sessions. We examine whether results differ for women and men by making matrices with themes and codes separately for both groups and inspecting these for subgroup differences. If feasible, we additionally search for patterns between other demographic characteristics, health situations, lifestyle behaviors, and financial situations (all measured using the questionnaire) and the themes and codes of the qualitative interviews. Additionally, descriptive statistical analyses are performed on the questionnaires. Finally, the lifelines are examined visually for recurring patterns over time and for relationships between difficulty making ends meet and CVD severity.

### Creative Work Sessions

Creative work sessions are organized using design thinking, drawing on principles of participatory research and co-design that facilitate the collaborative integration of participants’ experiences and insights in addressing complex challenges [37]. Its participatory and iterative nature is particularly valuable in citizen science as cocreation ensures that research tools and outcomes remain closely aligned with the experiences and perspectives of those involved [38]. In addition, these methods foster inclusive community engagement, actively involving underrepresented groups in the research process [39].

Within this project, design thinking is first applied in the co-design of a lifeline tool, developed together with the entire team to guide the interview process. The tool is collaboratively shaped to ensure that it provides a supportive structure for conducting the interviews and aligns with the interview topic list while also providing a supportive structure for the interviewers, helping them feel confident and facilitating both data collection and their comfort during data collection.

Following the creative and thematic analyses described above, creative work sessions are conducted to collaboratively explore ideas; discuss potential approaches; and iteratively generate recommendations for people, practice, and policy focused on the prevention and early detection of CVD among people experiencing financial hardship. In these work sessions, findings are collectively interpreted and synthesized using affinity mapping, clustering, and thematic synthesis. Citizen scientists and professionals play a key role in assessing the feasibility and relevance of these recommendations, ensuring that they reflect the perspectives and needs of the people themselves and the professionals who work with them. These sets of recommendations constitute the design output and will be presented in formats jointly determined by citizen and professional scientists, such as videos, infographics, exhibitions, or podcasts, with the aim of ensuring that the outputs are meaningful, relevant, and applicable in real-world contexts.

### Dynamic Learning Agenda

Throughout the project, the team collectively explores what is already known and what we still want or need to learn using a dynamic learning agenda that is adjusted based on current learning questions [40]. Learning questions can be answered in several ways, for example, by inviting experts to explain more about the topic, by assigning students to perform a literature review on the topic, by organizing training sessions, or through self-study.

### Evaluation of Collaboration

The collaboration and progress of our project team are evaluated approximately 6 or 7 times over the course of this project. During each evaluation round, individual interviews take place first, in which every citizen scientist is interviewed by a professional scientist and vice versa. Next, the most important points from these individual evaluation interviews are used as input for a group evaluation. Topics that are covered during the evaluation interviews include collaboration, personal development, challenges, and expectations. In addition, there is room for the interviewees to bring up their own topics that they would like to discuss and use as input for a group evaluation.

The goal of this continuous evaluation process is 2-fold. First, it allows for improvement in collaborating as a group, as various challenges may arise during the different phases of the project. It ensures that every member of the team can share their doubts, needs, and ideas for improvement. Second, it generates valuable data about citizen science as a method and how it is experienced by the different members of the group at different stages of the project.

### Ethical Considerations

Both the citizen scientists in the research team and the interviewees in the study belong to a potentially vulnerable group, which makes it particularly important to consider the ethical aspects of the study. Participation as citizen scientists and as interviewees is strictly voluntary. Reciprocity is an important principle in this study. Participation should not only benefit the research but also provide value to those involved. This means that citizen scientists are given opportunities to develop research skills, reflect on their own experiences, and contribute meaningfully to the research process. Interviewees are offered a space to share their stories in a respectful and safe setting, and their insights are used to inform concrete outcomes that may benefit the wider community. Participation in the study is confidential, with the identities (names and email addresses) of the interviewees known to only 2 of the professional scientists and the interviewer (ie, citizen scientist) for purposes of contacting individuals for participation in the interview. Interviewees will not be identifiable in any publications or other dissemination of research results. This will be explained to interviewees in the study information letter before they provide informed consent.

Interviewees will participate after providing written informed consent, and consent will be checked verbally before each interview. Consent is an ongoing process and is also checked during the interview if a participant becomes upset or talks about a particularly sensitive issue. After the qualitative interviews, aftercare is offered to both the citizen scientist interviewer and the interviewee if needed. This is provided by the professional expert by experience in the project team and, if necessary, a psychologist. For interviewers, there is also space during or after project meetings to talk about any triggering things said during interviews. Interviewees receive a voucher of €25 (US $29.51) for their time.

For the evaluation of the citizen science process, citizen scientists and professional scientists should feel safe to talk about other members of the project team when necessary. Therefore, everyone can choose with whom they want to do the evaluation interviews, and transcripts are anonymized. The Dutch code of conduct for academic integrity is explained during project meetings; the university integrity officer has been introduced, and team members are informed that any integrity concerns can be discussed with this officer.

The study received ethics approval from the ethics committee of Avans University of Applied Sciences (25.004) and the Ethics Committee of the Faculty of Health, Medicine, and Life Sciences at Maastricht University (FHML-REC/2025/049).

## Results

The study was funded in October 2024 and started in January 2025. Data collection started in November 2025 and is expected to end halfway through 2026. Four qualitative lifeline interviews had been conducted as of December 6, 2025. Data analyses are planned for 2026. Manuscripts reporting findings on the central research question and the process evaluations will be submitted for publication in 2027.

## Discussion

### Citizen Science With an Underrepresented Group

This research focuses on a group that often remains underrepresented in research: people who have difficulty making ends meet and, therefore, face an increased risk of CVD. By collaborating with citizen scientists who have personal experience with financial hardship and/or CVD, the study gains a unique perspective [41]. Their experiential knowledge ensures that the research resonates with the everyday realities of those living in these circumstances and that the findings and recommendations from the study will be more societally relevant. This cocreative approach not only enhances the relevance of the research questions and the practical applicability of the results but also fosters stronger connections with the people being interviewed, which could lead to more honest answers [41,42]. In addition, involving representatives from a group that is often underrepresented in research in a central role throughout the project may help empower the priority group and ensures that their perspectives are being heard and amplified [42-44].

A notable methodological feature of this study is the use of design thinking principles. Although increasingly applied in health research, their explicit use within citizen science remains relatively uncommon. Design thinking offers advantages in this context as it emphasizes the role of citizens, allows for meaningful engagement even with small groups, and generates tangible outputs that can be reviewed and adopted by the community [38]. However, applying this approach in potentially vulnerable settings, such as with people experiencing financial hardship, may present specific challenges. While design thinking typically involves structured planning of activities, it is anticipated that flexibility will be essential in this study to accommodate participants’ varying capacities, availability, and circumstances without compromising the objectives of the research.

### Challenges

Although we expect to encounter several challenges during the study, each of these also presents valuable opportunities for growth and improvement. For example, there is significant differentiation within the group of citizen scientists in terms of educational background, digital literacy, and research experience. This diversity requires accessible communication and clear agreements to ensure that everyone can participate equally [41,45]. By being transparent about the approach and working together on an equal footing, this project contributes to a more positive image of citizen science and reduced stigma regarding financial hardship.

Another challenge concerns the quality of the qualitative interviews, which are conducted by trained citizen scientists who are not professionally trained researchers [46,47]. While this may influence the scientific rigor of the interviews, it also allows participants to better relate to the interviewer and feel more comfortable sharing their stories [46,47]. There is sometimes skepticism about the quality of the data collected by citizen scientists [46]. However, research demonstrates that citizen scientists can gather data of quality to professional settings when proper training, protocols, and quality control measures are implemented [47].

A significant challenge that requires particular attention is the reality of health-related attrition among citizen scientists. Due to their (lived) experience with financial hardship and their own health problems or health crises among family members and close contacts, project team members regularly face periods when they cannot fully participate. This pattern of intermittent availability is, unfortunately, more prevalent in our project group as they experience higher rates of illness, hospitalization, social problems, and bereavement than people who do not have these risks. While experiential knowledge provides the project with invaluable insights and authenticity, it also means that we must accommodate the realities of these health and social challenges. To address this, our approach emphasizes maximum flexibility: team members can take a break when needed, can re-engage when their circumstances allow, and receive ongoing support from the project team [25]. We foster a culture of mutual understanding and support, recognizing that the health challenges that our citizen scientists face are inherently connected to the very issues that our research also seeks to address. This flexibility is not viewed as a limitation but as an essential ethical consideration when working with people in a vulnerable position in participatory research. By maintaining inclusive practices that accommodate varying levels of participation, we ensure that the voices of those affected by the intersection of financial hardship and CVD or other health issues remain central to our research even when their participation must be temporarily reduced.

Compensation and contracts for citizen scientists present additional complexities. Paying citizen scientists who receive social benefits is challenging in practice due to regulations in the Netherlands. Although volunteer contracts offer a solution, they come with limitations, such as a limit on the amount of compensation, restrictions on travel expense reimbursement, or having to prefinance travel expenses. In addition, these volunteer reimbursements are seen as expense claims and not as a salary by the university and, therefore, are not always paid on the same day of the month or an exact number of days since submitting the expense claim, causing additional financial stress for citizen scientists who already have difficulty making ends meet. This situation demands new procedures and customization from research organizations.

**Figure 1.**
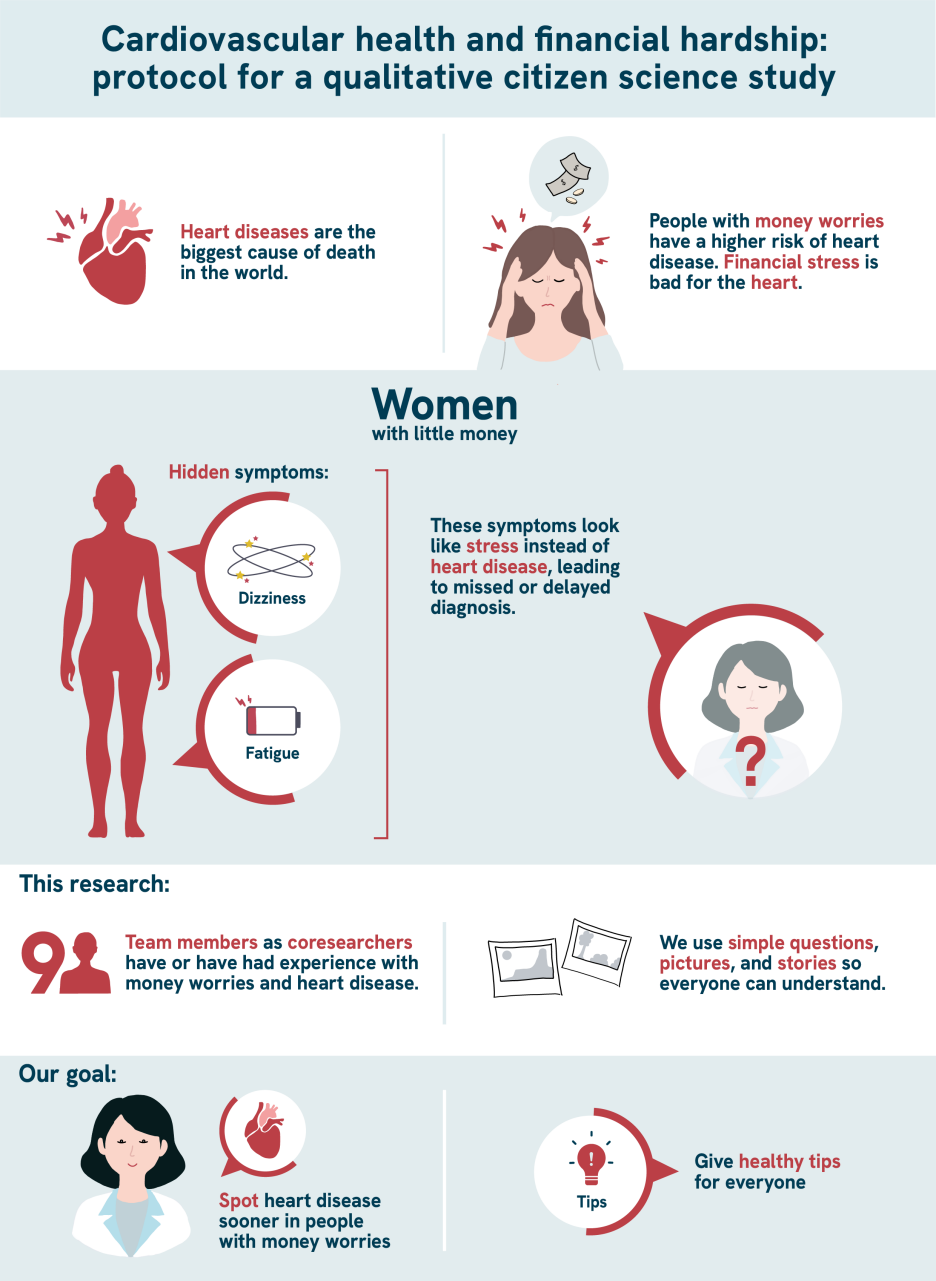
Visual summary of the study.

Finally, language use is essential in reducing stigma and disparities. When the language used resonates with the experiences of people, it fosters recognition and trust [41,45]. This connection is crucial for encouraging individuals to share their personal stories and for truly reaching those who might otherwise remain unseen [42,45]. Accessible language also increases the chances for everyone to understand all the information and participate actively, whether as research participants or as citizen scientists [41,45,48]. Therefore, it is important that clear and understandable language is used not only during the research process but also in final knowledge products such as articles and recommendations [41,48]. This approach ensures that findings are relevant to and usable by a broad audience, contributes to equal opportunities in health, and supports the goal of inclusive science [41,45]. For that reason, a visual summary of the study protocol has been added to this publication (Figure 1).

### Recommendations and Dissemination

Our study aims to examine multiple determinants of health considering not only medical factors but also lifestyle, mental health, living and working environment, and socioeconomic circumstances [49]. This broad approach leads to a better understanding of the complex challenges that people face. Additionally, this approach will help in formulating recommendations for various stakeholders (people who have difficulty making ends meet themselves, health care and social professionals, health promoters, and policymakers) to prevent CVD and recognize CVD earlier among people having difficulty making ends meet.

The process and results of our study are disseminated in a joint, participatory, and accessible manner [42,50]. For example, a visual summary of the study protocol has been added to this publication (Figure 1), and an accessible website was made in cocreation with citizen and professional scientists and a website designer [51]. Dissemination is conducted through open access academic articles, blogs, practical articles in Dutch, social media posts, media interviews, and recommendations for policy and practice involving both citizen and professional scientists in the process. The advisory committee also plays a role in increasing impact through disseminating our work in their professional networks. This approach not only builds and shares knowledge but also works to strengthen the position of people with experiential knowledge [42,43,49].

## Supplementary material

10.2196/89101 Peer Review Report 1 Peer review report by the Netherlands Organization for Health Research and Development.

## Abbreviations

CVD: cardiovascular disease
SEP: socioeconomic position
