# Supplementary material for: Cardiovascular Health and Financial Hardship: Protocol for a Qualitative Citizen Science Study
Source: JMIR Res Protoc. 2026 Mar 13;15:e89101. doi: 10.2196/89101 (PMC12986785; doi:10.2196/89101)
Supplement: Peer Review Report 1 [file resprot-v15-e89101-s001.pdf]

Start referentbeoordeling - Samen aan de hartSlag 2024

Formulier Referentenbeoordeling NL

1. Details subsidieaanvraag

1.1	Details subsidieaanvraag
**Subsidieronde:**	Citizen Science voor Gezondheid en Zorg
**Dossiernummer:**	04270042410003
**Aanvrager:**	Gera Nagelhout
**Projecttitel:**	Hart- en vaatziekten verminderen bij mensen die moeite hebben met rondkomen: een burgeronderzoek
**Code rapport:**	21391491

1.2 Code omgang met persoonlijke belangen en vertrouwelijkheid

Als u akkoord gaat met de beoordeling van deze aanvraag, gaat ZonMw ervan uit dat u als onafhankelijk deskundige op het betreffende vakgebied kunt worden aangemerkt, zoals bedoeld in de Code omgang met persoonlijke belangen van ZonMw. Mocht dit niet het geval zijn, meld dit dan zo snel mogelijk bij ZonMw. Neem bij twijfel hierover contact met ons op.

In onze procedure is vastgelegd dat de gehele beoordelingsprocedure een strikt vertrouwelijk karakter heeft. Dit betekent dat wij de referentoordelen altijd anonimiseren, maar ook dat u de subsidieaanvragen strikt vertrouwelijk dient te behandelen. Daarom mogen deze documenten niet worden gebruikt als input voor generatieve AI-modellen. Bovendien mag bij het opstellen van de beoordeling op geen enkele wijze gebruik gemaakt worden van generatieve AI-modellen. Meer informatie hierover vindt u op de ZonMw website.

- ☑ Ik verklaar een onafhankelijke expert te zijn zonder aanmerkelijk belang
- ☑ Ik verklaar dat ik de inhoud van de subsidieaanvraag en alle aanvullende informatie in dit beoordelingsproces strikt vertrouwelijk zal houden.
- ☑ Ik verklaar in deze beoordeling geen gebruik te maken van generatieve AI-modellen.

Uw beoordeling levert een belangrijke bijdrage aan de uiteindelijke beoordeling van de subsidieaanvraag en het besluit om de aanvraag wel of niet te honoreren. We zouden het dan ook zeer op prijs stellen als u onderstaande vragen zo gedetailleerd mogelijk beantwoordt.

1.3 Subsidieoproep

Voorafgaande aan het beoordelen van deze aanvraag vragen wij u de subsidieoproep goed door te lezen. Houd deze als leidraad bij de hand bij het beoordelen van de aanvraag.

- **Subsidieoproep**

2. Beoordeling

2.1 Probleemstelling, doelstelling en/of taakstelling

Beoordeel de kwaliteit van de doelstelling(en) en de aansluiting op de probleemstelling.

- **De probleemstelling is helder en concreet geformuleerd.**
- **De doelstelling van het onderzoeksproject is duidelijk beschreven. De doelstelling is helder en maakt duidelijk wat de aanvragers willen bereiken.**
- **Het kennisdoel (welke kennis willen ze verzamelen?) en het veranderdoel (welke verandering willen ze bereiken?) is duidelijk.**
- **De doelstelling sluit aan op het ingebrachte onderwerp van de burgerwetenschapper.**
- **De werkwijze/taakstelling sluit aan bij de doelstellingen van het project.**
- **De te behalen mijlpalen sluiten aan bij de doelstelling(en) van het project.**

**Score:**	Zeer goed

Licht hier uw oordeel toe.
Geef uw overwegingen aan en benoem daarbij positieve en negatieve punten (elk maximaal 4000 karakters)

Positieve punten:

Het probleem, meer inzicht krijgen welke factoren aanleiding geven tot hart en vaatziekten bij de doelgroep van mensen die moeilijk rondkomen is een belangrijke opdracht om tot een advies te komen. Om daarbij gebruik te maken van een onderzoeksgroep die bestaat uit beroepswetenschappers en burgerwetenschappers is in dit geval heel relevant om geloofwaardige interviews te kunnen afnemen. De data worden op verschillende manieren verzameld en geanalyseerd, met meer conventionele en minder conventionele methoden, wat een genuanceerd beeld zal geven. De verschillende groepen die betrokken zijn bij dit onderzoek maakt het heel divers en dus inclusief, wat de kwaliteit ten goede zal komen. Het onderzoek zal zeker een uitkomst opleveren, wat tot concrete aanbevelingen zal leiden. In een vervolg onderzoek kan dan bestudeerd worden wat de impact daarvan is, en dat kan potentieel groot zijn als we kijken naar het aantal mensen dat leeft in armoede (met hart en vaatziekten). De burgerwetenschappers worden zeer goed benut en dat is een grote kracht van dit voorstel. Na het bereiken van het kennisdoel, het identificeren van de grootste risicofactor(en) voor HVZ bij mensen die moeilijk rondkomen, komt het omzetten tot een veranderdoel, het vormen van adviezen, vanzelf aan de beurt.

Negatieve punten:

Enkele aspecten in de methoden zijn me nog niet helemaal duidelijk en komen later aan bod. Onderzoek doen is geen hobby en of de uitkomsten van een onderzoek representatief zijn staat of valt bij de kwaliteit van de uitvoering ervan en dus van de begeleiding/voorbereiding van de mensen die de interviews zullen afnemen.

2.2 Plan van aanpak (implementatieplan, projectbeschrijving)

Beoordeel het plan van aanpak van het project op basis van onderstaande criteria.

- **Het plan van aanpak is helder en concreet beschreven.**
- **Het plan van aanpak is duidelijk, haalbaar en geschikt om de doelstelling te behalen. Er is duidelijk beschreven hoe kennis ontwikkeld wordt en hoe een verandering wordt bereikt.**
- **Er is een duidelijke beschrijving en onderbouwing van de gekozen aanpak (methoden) en onderzoek (analyses) om de doelen te bereiken.**
- **Uit het plan van aanpak blijkt dat het onderzoeksproject door burgerwetenschapper en beroepswetenschapper samen uitgevoerd wordt. Er is hierbij duidelijk beschreven hoe de gelijkwaardige samenwerking tussen burger- en beroepswetenschappers eruit zal zien.**
- **Er zijn concrete implementatiestrategieën en -activiteiten opgenomen in het plan. Deze sluiten aan bij de doelstellingen.**
- **Er worden in het plan duidelijk stappen beschreven hoe de borging in de praktijk te garanderen. De onderdelen en/of fasen zijn concreet beschreven.**
- **De resultaten van het project worden op passende wijze geëvalueerd.**

**Score:**	Goed

Licht hier uw oordeel toe.
Geef uw overwegingen aan en benoem daarbij positieve en negatieve punten (elk maximaal 4000 karakters)

Positieve punten:

Over het algemeen is het een heel aantrekkelijk voorstel, maar ik heb nog wel een aantal vragen die ik bij onderstaande negatieve feedback zal beschrijven. De samenwerking tussen beroeps en burger wetenschappers lijkt heel gelijkwaardig, wat een grote plus is. Een adviescommissie met andere expertise is heel nuttig om te zorgen dat alles goed is doordacht en uitgewerkt voordat er begonnen wordt met de interviews.

Negatieve punten:

Interviews afnemen op een neutrale manier zonder beïnvloeding is best een kunst. Hoe wordt ervoor gezorgd dat de burgerwetenschappers dit in voldoende mate beheersen? Namelijk als dit niet goed gebeurt zijn de uitkomsten van het onderzoek niet betrouwbaar en meer een reflectie van de relatief kleine groep burgerwetenschappers ipv het cohort geïnterviewden.
Er wordt een aantal keer gerefereerd dat er al vrij veel kwantitatieve data is. Wordt daarmee bedoeld de gestandaardiseerde (verkorte) testen zoals

Quality of life tests (zoals SF12), of CIS of HADS testen zoals gebruikt vanuit de psychologie? Daar zitten namelijk meetbare elementen in die ook al richting geven. Wordt er naast de kwalitatieve tests dus geen enkele gestandaardiseerde meetbare test meegenomen omdat die allemaal al zijn uitgevoerd in deze doelgroep? Het leek mij juist handig als er wel 1 soort test op z'n minst wordt afgenomen om zo gekwantificeerd te hebben dat het nieuwe studie-cohort inderdaad problemen ervaart (tov de gehele Nederlandse populatie/andere controle groep uit literatuur waar genoeg data voor is om mee te vergelijken).
Wat betreft de literatuur studiegroepen begrijp ik dat de wetenschappelijke literatuur wordt benut, met name reviews die overzicht geven over het veld en een soort waardering/validatie van de verschillende studies geven die aan een bepaalde kwaliteit voldoen en dus hoeveel waarde aan bepaalde studies gehecht dient te worden. Maar wat betreft de niet wetenschappelijke studies over blogs of wat in de media aandacht kreeg vraag ik mij af hoe daar een keuze wordt gemaakt in wat een studie/blog/artikel is dat aandacht/tijd verdient om te worden besproken en wat niet. Is het doel om te kijken wat er onder de burgerwetenschappers leeft en wordt opgepikt om dat te ontzenuwen en inzicht te geven in de zin en onzin die overal te lezen is (want voor iedereen is een artikel te vinden online dat in zijn/haar denk-straatje past)? Of is het doel om te kijken waar de doelgroep op aanslaat als het om media gaat, zodat er betere media content gemaakt kan worden als we weten hoe men het gepresenteerd wil hebben? Want naar dat laatste zijn vast al andere studies gedaan (door psychologen of communicatie wetenschappers?) en dan zou ik aansluiting zoeken bij mensen die dit soort onderzoeken begeleiden, dat die onderzoekers meedoen in deze literatuur gesprekken? Dus wat is eigenlijk het doel van deze literatuur sessies? Dat kan duidelijker en dan wellicht meer benut worden.
De "photovoice" klinkt interessant, maar ik zou dan niet voor vrijblijvendheid kiezen, maar zorgen dat er bij ieder interview minstens 1 foto wordt gemaakt, zodat dit echt meegenomen kan worden als aparte analyse. De vrijheid zit m dan in wat de interviewer of de geïnterviewde fotografeert. (Een compleet zwarte foto is ook informatie.)
Hoe is men tot een groepsgrootte gekomen van 60 (30m/30v) interviews? Is dat op basis van ervaring met eerdere soortgelijke onderzoeken, waaruit bleek dat dit representatief genoeg is? Is dit een tijdkwestie, dat er anders niet genoeg uitwerk/analyse tijd over blijft? Als er wel 100 mensen gevonden kunnen worden, is dit dan te overwegen om zoveel mensen te includeren? Of hoe gaat men dan om met inclusie, first come first serve?
"Beveiligde opslag voor ten minste 15 jaar na de laatste publicaties van het project. " Wat gebeurt er na die 15 jaar? Wordt de data dan vernietigd of automatisch openbaar? Wat is daar gebruikelijk?

2.3 Samenwerking (partnerschap, projectgroep, netwerk)

Beoordeel de samenwerking binnen het project op basis van onderstaande criteria.

- **Alle leden van de projectgroep zijn beschreven.**
- **De rol van de burger in het project als (mede-)projectleider en/of (mede-) onderzoeker is duidelijk beschreven.**
- **Er is beschreven waarom de projectgroepleden geschikt zijn als deelnemer aan het project. Denk aan expertise en motivatie om het project succesvol te kunnen uitvoeren:**
- **De burger heeft zelf ervaring met het onderwerp of staat dichtbij burgers die zelf ervaring hebben met het onderwerp.**
- **De beroepswetenschapper heeft ervaring met burgerwetenschap of participatief onderzoek.**
- **Zowel de burger als de beroepswetenschapper zijn gemotiveerd om samen te werken, flexibel en open om te leren.**
- **Er is beschreven hoe er aandacht is voor een goede samenwerking. Iedereen moet zich veilig, gehoord en gezien voelen. Ook is beschreven hoe er aandacht is voor een leerproces van iedereen in de projectgroep.**

**Score:**	Zeer goed

Licht hier uw oordeel toe.
Geef uw overwegingen aan en benoem daarbij positieve en negatieve punten (elk maximaal 4000 karakters)

Positieve punten:

Die verschillende groepen zijn erg goed beschreven met hun rolverdeling. Daaruit voort vloeien verschillende netwerken die ingezet kunnen worden voor verschillende onderdelen van het project als het gaat om inclusie van mensen om te interviewen of het uitwerken van data of het maken van adviezen. Er is een significante en gelijkwaardige rol voor de burgerwetenschappers weggelegd. De individuen zijn goed beschreven en geschikt voor het onderzoek qua profiel. Ervaring met dit type onderzoek is ingebed, mede door het samenvoegen van de 2 losse projectaanvragen in deze constructie. Het klinkt als een mooi team waarin veiligheid gewaarborgd is door op verschillende momenten ook binnen het team na te gaan hoe iedereen in het project zit. En er is extra hulp beschikbaar aan de zijlijn mocht dit aan de orde zijn.

Negatieve punten:

Het is een vrij groot team van verschillende mensen, dus qua aansturing is het nodig dat dit overzichtelijk blijft voor de burgerwetenschappers en dat zij ook goed gemonitord blijven gedurende de onderzoeksperiode.

2.4 Haalbaarheid

Beoordeel de haalbaarheid van het project op basis van de onderstaande criteria:

- **Er is een duidelijke tijdsplanning voor het project met een maximale looptijd van het onderzoeksproject van 30 maanden.**
- **Er is duidelijk gemaakt dat het plan van aanpak haalbaar is. En dat het lukt om het doel van de subsidieaanvraag met de projectleden binnen de tijd en binnen het budget te bereiken.**
- **Maak duidelijk dat het project binnen 6 maanden nadat je gehoord hebt dat het project is goedgekeurd kan starten.**

**Score:**	Goed

Licht hier uw oordeel toe.
Geef uw overwegingen aan en benoem daarbij positieve en negatieve punten (elk maximaal 4000 karakters)

Positieve punten:

Het project kan zo gestart worden en zal qua budget ook prima uitkomen.
**Negatieve punten:**
Hoe snel de inclusie gaat en al die verschillende vormen van data verwerking en analyses gaan uitpakken blijft een beetje een vraag. Uiteraard wordt een planning gegeven, maar hier zit een onzekere factor. Dus goed monitoren en tijdig bijspringen voor data verwerking moet gewaarborgd worden.

2.5 Impact: kennisoverdracht, kennisbenutting, en kennisborging

Beoordeel in de aanvraag de mate waarin kennis en kunde wordt gedeeld, benut en geborgd in praktijk, beleid, onderwijs en/of onderzoek. Doe dit op basis van de onderstaande criteria:

- **Het is duidelijk welke resultaten worden verwacht bij het project en voor wie die van belang zijn.**
- **De resultaten en de toepassing zijn nuttig voor anderen. Activiteiten die worden bedacht voor de verspreiding van de resultaten (opgedane kennis en ervaringen) en de toepassing ervan zijn beschreven.**
- **Het is duidelijk gemaakt op welke wijze de resultaten uit het project geborgd gaan worden in de praktijk.**
- **Resultaten worden laagdrempelig gedeeld met een breed publiek. Ook creatieve manieren om dit te doen worden hierbij gewaardeerd.**

**Score:**	Zeer goed
**Licht hier uw oordeel toe.<br>Geef uw overwegingen aan en benoem daarbij positieve en negatieve punten (elk maximaal 4000 karakters)**	
**Positieve punten:**	
Dat het onderzoek relevant is is compleet helder. Er worden allerlei manieren beschreven om de data bij verschillende doelgroepen onder de aandacht te brengen zoals bij de mensen die moeilijk rond komen zelf, maar ook bij zorgprofessionals, overheidsinstanties en andere onderzoekers, via conservatieve en moderne media. Dus aan aandacht zal vast geen gebrek zijn. Het is ook een onderzoek dat tot de verbeelding spreekt dus het leent zich goed voor media aandacht en de impact kan dus heel groot zijn en hopelijk iets teweeg brengen.	
**Negatieve punten:**	
Naast de bestaande beschreven kennis overdracht kan ook nog worden nagedacht over een wervende actie die het uitrollen van de adviezen mogelijk kan maken. Maar dit zal tzt vast bedacht worden samen met de Hartstichting.	

2.6 Samenvattend kwaliteitsoordeel

Geef een samenvattend kwaliteitsoordeel over de aanvraag op basis van bovenstaande onderdelen.

**Score:**	Zeer goed
**Licht hier uw oordeel toe.<br>Geef uw overwegingen aan en benoem daarbij positieve en negatieve punten (elk maximaal 4000 karakters)**	
**Positieve punten:**	
Het is een pijnpunt in onze samenleving dat mensen met moeite met rond komen meer last hebben van allerlei ziekten waaronder HVZ, dat de grootste bedreiging is voor de nationale gezondheid en een epidemie aan het worden is. Dus dat we beter leren begrijpen wat hier allemaal speelt lijkt me heel nuttig en in deze vorm zoals beschreven in de aanvraag een zeer interessante aanpak. De uiteindelijke praktische adviezen nav dit onderzoek maken het hopelijk mogelijk om hier meer mee te doen dan tot nu toe is gelukt met de huidige methoden.	
**Negatieve punten:**	
Slechts enkele technische details die ik eerder heb beschreven.	

2.7 Begroting

Beoordeel de begroting op basis van de onderstaande criteria:

- **De begroting dient onderbouwd, redelijk en in overeenstemming te zijn met het plan van aanpak van de aanvraag.**
- **In de begroting staat duidelijk wie vanuit welke organisatie in welke functie voor hoeveel uur wordt ingezet.**
- **Er is beschreven hoe de burger een passende aanstelling of vergoeding krijgt voor de inzet die de burger gaat leveren.**
- **Er is geld gereserveerd voor vergoedingen voor mensen die flexibel of eenmalig betrokken worden.**
- **Er is minimaal 5% van het projectbudget gereserveerd voor het verspreiden en toepassen van de resultaten. Kosten van een communicatieadviseur vallen hier ook onder.**

**De begroting is:**	Realistisch

**Licht hier uw oordeel toe.**<br>**Geef uw overwegingen aan en benoem daarbij positieve en negatieve punten (elk maximaal 4000 karakters)**
**Positieve punten:**
Het is een hele realistische begroting, dus ik zie geen rare dingen.
**Negatieve punten:**
kan niks vinden

Start referentbeoordeling - Samen aan de hartSlag 2024

Formulier Referentenbeoordeling NL

1. Details subsidieaanvraag

1.1	Details subsidieaanvraag
**Subsidieronde:**	Citizen Science voor Gezondheid en Zorg
**Dossiernummer:**	04270042410003
**Aanvrager:**	Gera Nagelhout
**Projecttitel:**	Hart- en vaatziekten verminderen bij mensen die moeite hebben met rondkomen: een burgeronderzoek
**Code rapport:**	21391481

1.2 Code omgang met persoonlijke belangen en vertrouwelijkheid

Als u akkoord gaat met de beoordeling van deze aanvraag, gaat ZonMw ervan uit dat u als onafhankelijk deskundige op het betreffende vakgebied kunt worden aangemerkt, zoals bedoeld in de Code omgang met persoonlijke belangen van ZonMw. Mocht dit niet het geval zijn, meld dit dan zo snel mogelijk bij ZonMw. Neem bij twijfel hierover contact met ons op.

In onze procedure is vastgelegd dat de gehele beoordelingsprocedure een strikt vertrouwelijk karakter heeft. Dit betekent dat wij de referentoordelen altijd anonimiseren, maar ook dat u de subsidieaanvragen strikt vertrouwelijk dient te behandelen. Daarom mogen deze documenten niet worden gebruikt als input voor generatieve AI-modellen. Bovendien mag bij het opstellen van de beoordeling op geen enkele wijze gebruik gemaakt worden van generatieve AI-modellen. Meer informatie hierover vindt u op de ZonMw website.

- ☐ Ik verklaar een onafhankelijke expert te zijn zonder aanmerkelijk belang
- ☑ Ik verklaar dat ik de inhoud van de subsidieaanvraag en alle aanvullende informatie in dit beoordelingsproces strikt vertrouwelijk zal houden.
- ☑ Ik verklaar in deze beoordeling geen gebruik te maken van generatieve AI-modellen.

Uw beoordeling levert een belangrijke bijdrage aan de uiteindelijke beoordeling van de subsidieaanvraag en het besluit om de aanvraag wel of niet te honoreren. We zouden het dan ook zeer op prijs stellen als u onderstaande vragen zo gedetailleerd mogelijk beantwoordt.

1.3 Subsidieoproep

Voorafgaande aan het beoordelen van deze aanvraag vragen wij u de subsidieoproep goed door te lezen. Houd deze als leidraad bij de hand bij het beoordelen van de aanvraag.

- **Subsidieoproep**

2. Beoordeling

2.1 Probleemstelling, doelstelling en/of taakstelling

Beoordeel de kwaliteit van de doelstelling(en) en de aansluiting op de probleemstelling.

- **De probleemstelling is helder en concreet geformuleerd.**
- **De doelstelling van het onderzoeksproject is duidelijk beschreven. De doelstelling is helder en maakt duidelijk wat de aanvragers willen bereiken.**
- **Het kennisdoel (welke kennis willen ze verzamelen?) en het veranderdoel (welke verandering willen ze bereiken?) is duidelijk.**
- **De doelstelling sluit aan op het ingebrachte onderwerp van de burgerwetenschapper.**
- **De werkwijze/taakstelling sluit aan bij de doelstellingen van het project.**
- **De te behalen mijlpalen sluiten aan bij de doelstelling(en) van het project.**

**Score:**	Goed

Licht hier uw oordeel toe.
Geef uw overwegingen aan en benoem daarbij positieve en negatieve punten (elk maximaal 4000 karakters)

Positieve punten:

Probleem - en doelstelling zijn allereerst heel helder geformuleerd. Het kennis- en veranderdoel zijn duidelijk. Met name de deelvragen leveren daarvoor stapsgewijs de benodigde informatie op, en duiden daarnaast op het gelijkwaardig verweven van ervaringskennis met wetenschappelijke kennis. De gekozen groep, mensen die moeite hebben met rondkomen, met daarbinnen speciale focus op vrouwen, sluit aan op het ingebrachte onderwerp van twee burgerwetenschappartijen; de adviesgroep uit Maastricht en Dagmar Niewold als zelfstandige.

Negatieve punten:

Het daadwerkelijk voorkomen (primaire preventie) van HVZ bij mensen die moeite hebben met rondkomen is genoemd als primair doel, maar dat lijkt me helaas niet (volledig) haalbaar met de gestelde deelvragen en de beoogde op te halen data/informatie. (Ook omdat andere factoren zoals erfelijkheid een rol kunnen spelen)

2.2 Plan van aanpak (implementatieplan, projectbeschrijving)

Beoordeel het plan van aanpak van het project op basis van onderstaande criteria.

- **Het plan van aanpak is helder en concreet beschreven.**
- **Het plan van aanpak is duidelijk, haalbaar en geschikt om de doelstelling te behalen. Er is duidelijk beschreven hoe kennis ontwikkeld wordt en hoe een verandering wordt bereikt.**
- **Er is een duidelijke beschrijving en onderbouwing van de gekozen aanpak (methoden) en onderzoek (analyses) om de doelen te bereiken.**
- **Uit het plan van aanpak blijkt dat het onderzoeksproject door burgerwetenschapper en beroepswetenschapper samen uitgevoerd wordt. Er is hierbij duidelijk beschreven hoe de gelijkwaardige samenwerking tussen burger- en beroepswetenschappers eruit zal zien.**
- **Er zijn concrete implementatiestrategieën en -activiteiten opgenomen in het plan. Deze sluiten aan bij de doelstellingen.**
- **Er worden in het plan duidelijk stappen beschreven hoe de borging in de praktijk te garanderen. De onderdelen en/of fasen zijn concreet beschreven.**
- **De resultaten van het project worden op passende wijze geëvalueerd.**

**Score:**	Goed

Licht hier uw oordeel toe.
Geef uw overwegingen aan en benoem daarbij positieve en negatieve punten (elk maximaal 4000 karakters)

Positieve punten:

Het plan van aanpak is heel helder geformuleerd. Het onderzoeksgedeelte is uitermate geschikt om het kennisdoel te halen, en de deelvragen te beantwoorden. Bovendien is het geschikt om het kennisdoel te behalen met gelijkwaardigheid tussen beroeps- en burgerwetenschappers. Dit komt terug in de methode, maar vooral in de gekozen data analyse strategie. Het onderzoek is haalbaar en geschikt.

Negatieve punten:

Dit voorstel lijkt vooral te focussen op kennis. Voor wat betreft het veranderdoel is het plan van aanpak minder haalbaar en geschikt. Implementatiestrategieën zijn weinig concreet, borging in de praktijk wordt niet genoemd. Disseminatie-activiteiten lijken vooral op "zenden" te worden ingestoken, door middel van (praktische) artikelen, infographics, videos, blog. Ik mis in het plan wat de burgerwetenschappers zelf zien als een geschikt medium of kanaal. Wanneer zijn zij tevreden? Wat zouden ze graag zien op dit gebied?
Een blogbereik van 1500 jaarlijkse bezoekers lijkt me vrij beperkt.

Het veranderdoel van dit project is om:
1. mensen die moeite hebben met rondkomen te helpen om HVZ te voorkomen en eerder te herkennen.

2. professionals te leren om HVZ eerder te herkennen bij vrouwen en mannen die moeite hebben met rondkomen.
3. interventies gericht op het voorkomen van HVZ geschikter te maken voor mensen die moeite hebben met rondkomen.
Op de lange termijn zou dit ertoe kunnen leiden dat sociaaleconomische gezondheidsverschillen kleiner worden.

Deze doelen worden beoogd te halen met aanbevelingen, o.a. via een vaktijdschrift. Aanbevelingen alleen zijn niet genoeg om daadwerkelijk tot verandering te komen. Niet voor niets is er (relatief recent) een hele tak in de wetenschap gericht op implementatie. Design thinking wordt beschreven bij de creatieve werksessies en lijken uit de omschrijving vooral in te steken op diepere analyse. Design thinking kan ook worden ingezet voor het ontwerpen van verander-producten en -activiteiten. Ik zou aanraden om daar dan de partijen bij te betrekken bij wie je de verandering wil zien. Dus bijvoorbeeld de NHG, als je wil dat zij dit meenemen in richtlijnen. Ik begrijp dat de veranderingen zelf nog open liggen, maar het is wel duidelijk dat je daar verandering wil. En al is de verandering alleen maar dat men meer open staat voor het perspectief van burgerwetenschappers dan is dat al een positieve verandering). Wat ik hiermee bedoel, is dat er geen reden is om deze stakeholders niet nu al te betrekken. De begeleidingscommissie wordt aangesteld voor expertise die ontbreekt, en met name de cardiologen (met expertise in sekseverschillen) vormen inderdaad een relevante aanvulling, maar het zijn toch vooral ook beroepswetenschappers. Waarom is er niet iemand van de NHG uitgenodigd, of Zorgverzekeraars Nederland, of RIVM (Centrum Gezond Leven), VWS? Dan kun je meteen de implementatiestrategieën met hen vormgeven.

NB Toch de beoordeling goed, vanwege de zeer sterke kenniscomponent.

2.3 Samenwerking (partnerschap, projectgroep, netwerk)

Beoordeel de samenwerking binnen het project op basis van onderstaande criteria.

- **Alle leden van de projectgroep zijn beschreven.**
- **De rol van de burger in het project als (mede-)projectleider en/of (mede-) onderzoeker is duidelijk beschreven.**
- **Er is beschreven waarom de projectgroepleden geschikt zijn als deelnemer aan het project. Denk aan expertise en motivatie om het project succesvol te kunnen uitvoeren:**
- **De burger heeft zelf ervaring met het onderwerp of staat dichtbij burgers die zelf ervaring hebben met het onderwerp.**
- **De beroepswetenschapper heeft ervaring met burgerwetenschap of participatief onderzoek.**
- **Zowel de burger als de beroepswetenschapper zijn gemotiveerd om samen te werken, flexibel en open om te leren.**
- **Er is beschreven hoe er aandacht is voor een goede samenwerking. Iedereen moet zich veilig, gehoord en gezien voelen. Ook is beschreven hoe er aandacht is voor een leerproces van iedereen in de projectgroep.**

**Score:**	Goed

Licht hier uw oordeel toe.
Geef uw overwegingen aan en benoem daarbij positieve en negatieve punten (elk maximaal 4000 karakters)

Positieve punten:

Alle leden van de projectgroep, inclusief hun rollen, expertise en motivatie zijn beschreven.
De projectgroepleden hebben eerder samengewerkt en kennen elkaar dus.

Negatieve punten:

Het leerproces van iedereen en hoe daar aandacht voor is, is niet expliciet beschreven. Ik ben benieuwd wat de leerdoelen zijn van de beroepswetenschappers en van de burgerwetenschappers. Liggen die louter op het kennis/inhoudelijke vlak, of ook op het vlak van burgerwetenschap en samenwerking bijvoorbeeld? Sluit de interviewtraining aan bij de leerdoelen? (Ik neem aan van wel, maar het is niet expliciet gemaakt.)

2.4 Haalbaarheid

Beoordeel de haalbaarheid van het project op basis van de onderstaande criteria:

- **Er is een duidelijke tijdsplanning voor het project met een maximale looptijd van het onderzoeksproject van 30 maanden.**
- **Er is duidelijk gemaakt dat het plan van aanpak haalbaar is. En dat het lukt om het doel van de subsidieaanvraag met de projectleden binnen de tijd en binnen het budget te bereiken.**
- **Maak duidelijk dat het project binnen 6 maanden nadat je gehoord hebt dat het project is goedgekeurd kan starten.**

**Score:**	Goed

Licht hier uw oordeel toe.
Geef uw overwegingen aan en benoem daarbij positieve en negatieve punten (elk maximaal 4000 karakters)

Positieve punten:

Het plan van aanpak om het kennisdoel te halen is haalbaar. Er is ook nagedacht over eventuele risico's en hoe daarmee omgegaan zou kunnen worden.
Ethische toestemming wordt aangevraagd na goedkeuring, maar het is niet WMO-plichtig.

Negatieve punten:

Het veranderdoel is niet direct haalbaar, zie daarvoor de eerder gegeven comments. Er zijn geen echte implementatiestrategieen, alleen aanbevelingen, en er is geen plan om actoren te betrekken voor implementatie.

NB toch het oordeel goed, vanwege het zeer sterke plan voor het kennisdoel.

2.5 Impact: kennisoverdracht, kennisbenutting, en kennisborging

Beoordeel in de aanvraag de mate waarin kennis en kunde wordt gedeeld, benut en geborgd in praktijk, beleid, onderwijs en/of onderzoek. Doe dit op basis van de onderstaande criteria:

- **Het is duidelijk welke resultaten worden verwacht bij het project en voor wie die van belang zijn.**
- **De resultaten en de toepassing zijn nuttig voor anderen. Activiteiten die worden bedacht voor de verspreiding van de resultaten (opgedane kennis en ervaringen) en de toepassing ervan zijn beschreven.**
- **Het is duidelijk gemaakt op welke wijze de resultaten uit het project geborgd gaan worden in de praktijk.**
- **Resultaten worden laagdrempelig gedeeld met een breed publiek. Ook creatieve manieren om dit te doen worden hierbij gewaardeerd.**

**Score:**	Voldoende

Licht hier uw oordeel toe.
Geef uw overwegingen aan en benoem daarbij positieve en negatieve punten (elk maximaal 4000 karakters)

Positieve punten:

De kennisresultaten zijn duidelijk, evenals het belang en het nut.
Er worden creatieve sessies gepland om te duiden wat de resultaten betekenen.

Negatieve punten:

Het is niet duidelijk hoe resultaten geborgd gaan worden in de praktijk.

Het delen van resultaten wordt gedaan in de vorm van aanbevelingen maar is dat genoeg om tot verandering te komen (implementatie), laat staan borging (het langdurig volhouden), als de bewuste actoren die moeten veranderen niet aanwezig zijn?

De begeleidingscommissie wordt betrokken bij het kiezen van een vorm voor de professionals. Echter, er zitten (weinig tot) geen professionals in die commissie. De cardiologen zouden wellicht zo gezien kunnen worden, maar de groep professionals voor primaire en secundaire preventie is veel breder. Ik denk niet dat een cardioloog kan bijdragen over verandering in een NHG-richtlijn. Of dat zij het werk van POH-ers kunnen veranderen. Etc. Er zitten vooral beroepswetenschappers in die commissie, wat neer zou komen op het bedenken van aansluiting in de praktijk vanuit de ivoren toren.

2.6 Samenvattend kwaliteitsoordeel

Geef een samenvattend kwaliteitsoordeel over de aanvraag op basis van bovenstaande onderdelen.

**Score:**	Goed

Licht hier uw oordeel toe.
Geef uw overwegingen aan en benoem daarbij positieve en negatieve punten (elk maximaal 4000 karakters)

Positieve punten:

Ik geef ondanks de eerder genoemde opmerkingen het kwaliteitsoordeel goed, omdat er veel goed doordachte elementen aan dit zeer begrijpelijk geschreven voorstel zitten.

Negatieve punten:

Het is vooral gericht op kennis, en minder op verandering (implementatie, borging). Ik denk echter dat de voorgestelde projectstructuur en plan van aanpak voldoende mogelijkheden biedt, om wel meer kansen voor deze verandering te scheppen. Bijvoorbeeld door het uitbreiden van de begeleidingscommissie met professionals (en iemand vanuit patiëntperspectief ? bijv. patientenfederatie).

2.7 Begroting

Beoordeel de begroting op basis van de onderstaande criteria:

- **De begroting dient onderbouwd, redelijk en in overeenstemming te zijn met het plan van aanpak van de aanvraag.**
- **In de begroting staat duidelijk wie vanuit welke organisatie in welke functie voor hoeveel uur wordt ingezet.**
- **Er is beschreven hoe de burger een passende aanstelling of vergoeding krijgt voor de inzet die de burger gaat leveren.**
- **Er is geld gereserveerd voor vergoedingen voor mensen die flexibel of eenmalig betrokken worden.**
- **Er is minimaal 5% van het projectbudget gereserveerd voor het verspreiden en toepassen van de resultaten. Kosten van een communicatieadviseur vallen hier ook onder.**

**De begroting is:**	Realistisch
**Licht hier uw oordeel toe.**<br>**Geef uw overwegingen aan en benoem daarbij positieve en negatieve punten (elk maximaal 4000 karakters)**	
**Positieve punten:**	
50% kosten voor burgerwetenschappers, 50% beroepswetenschappers<br>Goed dat burgerwetenschapper en mede-projectleider een markt-conform uurtarief krijgt!<br>Het geheel lijkt realistisch, voor zover inzichtelijk is wat posten inhouden (zie onder).	
**Negatieve punten:**	
De additionele toelichting is niet helemaal leesbaar.<br>Het is niet duidelijk hoe groot en lang de aanstelling van de junior onderzoeker bij Avans is, en de onderzoeksassistent bij UM. Dat maakt het lastiger inschatten of het passend is.<br>Ook de eigen bijdragen van lector Avans en assistant professor zijn alleen in totaalbedragen, maar niet inzichtelijk in hoeveel tijd daadwerkelijk zal worden besteed.	
